# Monotonic and Fatigue Behavior of EBM Manufactured Ti-6Al-4V Solid Samples: Experimental, Analytical and Numerical Investigations

**DOI:** 10.3390/ma13204642

**Published:** 2020-10-17

**Authors:** Wiebke Radlof, Christopher Benz, Horst Heyer, Manuela Sander

**Affiliations:** Institute of Structural Mechanics, Faculty of Mechanical Engineering and Marine Technology, University of Rostock, 18059 Rostock, Germany; christopher.benz@uni-rostock.de (C.B.); horst.heyer@uni-rostock.de (H.H.); manuela.sander@uni-rostock.de (M.S.)

**Keywords:** electron beam melting, Ti-6Al-4V, monotonic and cyclic stress-strain behavior, strain-life test, Johnson–Cook failure model, Ramberg–Osgood model, Coffin–Manson model

## Abstract

The present study aims to carry out an experimental, analytical and numerical investigation of the monotonic and fatigue performance of electron beam melted Ti-6Al-4V structures. Therefore, tensile tests, multiple step tests and strain-life tests were performed on machined EBM Ti-6Al-4V solid samples. An elastic-plastic material model in combination with a numerical damage model was examined according to the experimental tensile tests. Analytical models proposed by Ramberg and Osgood, as well as Coffin and Manson were obtained to describe the cyclic stress-strain curves and strain-life curves, respectively. The fracture surfaces of the tested samples and the influence of different build directions were analyzed. A prediction of the static and fatigue material properties is of particular importance, e.g., for the safe application of additively manufactured load-bearing implant structures. Based on the determined analytical and numerical models, the material and product behavior of complex electron beam melted structures under cyclic loading and fatigue life determination can be investigated in the early stages of the product development process.

## 1. Introduction

Titanium alloys, such as Ti-6Al-4V, are the preferred material for applications in the biomedical context, due to their high strength-to-weight ratio, good corrosion resistance and excellent biocompatibility [1,2]. Because of its design freedom, additive manufacturing (AM) plays an important role in implant applications and offers numerous possibilities for the production of geometrically complex structures [3]. However, for a safe application of load-bearing implants, complex simulations of material and product behavior must be performed during the early stages of the product development process. Since load-bearing implants, such as hip implants, are loaded not only by monotonic, but also by cyclic loads, the precise characterization of the cyclic and fatigue properties of the additively manufactured material is as important as their monotonic properties.

For the fatigue design of load-bearing implants mainly stress-life approaches are applied in the literature. Thus, a number of studies determined the stress versus number of cycles to failure (S-N curves) and endurance limits associated with high-cycle fatigue (HCF) or very high-cycle fatigue (VHCF) [4,5,6,7,8,9,10]. However, the local strain-based approach, which is of great importance for complex AM metallic lattice structures, is not investigated in terms of electron beam melted (EBM) Ti-6Al-4V samples. However, investigations of the fatigue behavior of EBM Ti-6Al-4V structures have shown that process conditions, specifically the build orientation [11,12,13] and post-process heat treatments [14,15], as well as defects [8,16,17,18] and machining [4], are influencing factors. The results show that for example defects are generally inherent to the AM process. They act as micro-notches and cause stress concentrations [19]. Especially the micro-notch effect of the surface roughness was investigated in the literature [11]. Since the surface of additive manufactured implant structures is often post-machined, machined and polished samples are examined in this study, to characterize the material itself. Additively processed Ti-6Al-4V shows two distinct failure modes, i.e., surface and internal fatigue crack initiation [16]. These cracks initiate due to one of the two common EBM defect types, “porosity” and “lack of fusion” [4,16,18]. A generally valid statement about the influence of internal defects is very difficult, since many influencing factors, such as the defect location, orientation to the load direction, shape, density and size, must be involved. In the high cycle fatigue regime Murakami’s approach has recently been adopted in several studies to estimate the effect of defects in AM materials for use in fatigue life predictions [16,20,21] and has provided promising results [10,22,23]. 

In order to simulate the structural behavior of AM structures under static loads including large plastic deformations, such as the post-buckling behavior of axially compressed structures or ductile failure, true stresses and strains are necessary. Whereas the monotonic properties of EBM Ti-6Al-4V solid structures have been extensively studied in the literature [24,25,26,27,28], the derivations of true stresses and strains in the post-uniform elongation range are still missing. However, these are necessary for nonlinear numerical simulations in the framework of an updated Lagrange formulation. Some studies use elastic-plastic material data from EBM Ti-6Al-4V provided by the powder manufacturer (Arcam AB) [29] or determined from experiments [30], also in combination with the Johnson–Cook (J-C) failure model to predict the failure behavior of the investigated EBM manufactured structures [30]. However, a detailed experimental investigation of true stresses and strains as well as the determination of the J-C failure model and parameters taking the different build directions into account is not presented in the literature. 

To close this gap, this study investigates the monotonic and fatigue behavior of EBM manufactured Ti-6Al-4V solid structures experimentally, analytically and numerically. Monotonic tensile tests were performed in order to examine true stress-strain data and to develop a numerical damage model. Multiple and incremental step tests as well as strain-life tests were carried out to determine cyclic stress-strain and strain-life fatigue curves, respectively. For the analytical description of the determined curves the cyclic Ramberg–Osgood and Coffin–Manson approaches were applied. The build direction was considered in all performed experiments. Moreover, detailed fractographic analyses of the fracture surfaces were performed to provide a deeper understanding of underlying fracture mechanisms.

## 2. Materials and Methods 

### 2.1. Sample Design and Manufacturing 

**Figure 1:**
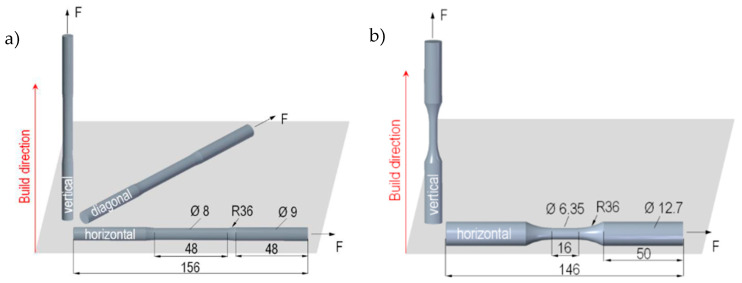
Specimen design in mm and build directions for (**a**) monotonic tensile tests and (**b**) fatigue tests.

For the quasi-static tensile tests, three different build directions (vertical, diagonal, horizontal) were considered. The specimen geometry was designed based on ISO 6892-1 standard [31] (Figure 1a). The geometry of the fatigue specimens with two different build directions (vertical, horizontal) were based on the ASTM standard E606 [32] (Figure 1b). The specimens were machined into the final geometries from cylindrical bars, which were additively manufactured by the electron beam melting (EBM) process with the same process parameters using an ARCAM A1 machine at the Institute of Microfluidics at the University of Rostock. The chemical composition of the used Ti-6Al-4V ELI powder is listed in Table 1.

### 2.2. Experimental Set-Up

Quasi-static tensile tests were carried out based on the international standard ISO 6892-1 [31] at room temperature. The tests were performed with a cross head speed of 0.01 mm/s. From the generated force displacement data, engineering stress-strain data were calculated. Moreover, mechanical properties including yield strength, ultimate tensile strength, Young’s modulus and elongation at fracture by taking three different build directions into account were determined.

Three types of strain-controlled fatigue experiments with a stress ratio of *R* = −1 were performed at room temperature using the servohydraulic testing machine Instron 8801. In multiple step tests (MST) the specimens were cycled with predefined strain amplitudes. These strain amplitudes were kept constant for 20 cycles and increased from 0.2% to 1.2% in 0.2% steps. Afterwards, the strain amplitudes were decreased in 0.1% steps from 1.2% to 0.2% with 10 cycles in each step to investigate interaction effects. In incremental step tests (IST), a cyclic loading was applied in blocks of about 20 cycles with increasing and decreasing strain amplitudes. Strain-based fatigue tests in terms of a multi-sample technique were performed to identify strain-life curves. For this, strain amplitudes between 0.3% and 1% were chosen. The samples were cycled at the defined strain amplitudes until failure or a total number of cycles of $5\times 10^{6}$ was reached. Two specimens were tested at one strain amplitude. A third specimen was chosen if the number of cycles to failure differed by over 40 percent. The test frequency of the fatigue tests was varied between 0.5 and 5 Hz, depending on the strain amplitude in order to avoid heating of the specimen. The experimental program for the conducted tests is summarized in Table 2.

On the one hand, the strain measurement was carried out using an extensometer with a gauge length of 40 mm ± 20 mm for static tests and 15 mm ± 2 mm for fatigue tests. On the other, the digital image correlation (DIC) technique was applied. The DIC technique was integrated into the test rig of the tensile tests for a contactless local strain analysis. Therefore, a camera system with a resolution of 12 megapixels was installed in the experimental set-up. The camera (Grasshopper®3, FLIR^®^ Systems, Inc.,Wilsonville, OR, USA) was connected to the DIC system, which enables an automatic triggering according to the intervals specified in the testing procedure. For tensile tests, images were recorded with a frame rate of 5 fps. In addition, an analog interface allows synchronizing the images to specific load values. Halogen spotlights ensured a proper illumination of the specimen surface. For this, an artificial speckle pattern was applied to the specimen surface with a black speckle on the previous white sprayed surface. Analysis of the recorded images was done using VIC-2D (Correlated Solutions Inc., Irmo, CA, USA). A step size of 3 pixels and a subset size of 29 pixels × 29 pixels were used. For a suitable evaluation of the fracture strain immediately prior to rupture the major principal strain (e1) was analyzed.

Fractography analyses were accomplished by digital microscopy using a Keyence microscope (VHX-5000, KEYENCE DEUTSCHLAND GmbH, Neu-Isenburg, Germany) to investigate the fracture surface of monotonic and fatigue samples. 

### 2.3. Analytical and Numerical Models

#### 2.3.1. Analytical Monotonic Stress-Strain Relation

In the following, the focus is on determining true stress-strain relations from engineering stress-strain curves of the tensile tests on machined hourglass specimens (Figure 1a). In the range of uniform deformations, the true stress-strain relations are analytically obtained. The true strains are calculated with

(1) $$\varepsilon =\ln\left(1+\varepsilon_{e}\right)$$

from the engineering strains $\varepsilon_{e}$ and the true stresses were determined using

(2) $$\sigma =\sigma_{e}\left(1+\varepsilon_{e}\right)$$

whereby $\sigma_{e}$ are the engineering stresses. After the onset of necking a linear continuation of the last slope is chosen

(3) $$\sigma =\sigma_{u}\left(1+\varepsilon_{e}-\varepsilon_{u}\right)$$

where the stress $\sigma_{u}$ and the strain $\varepsilon_{u}$ correspond to the true stress-strain data at the onset of necking. This generates stresses and strains for an approximately damage-free material response. Damage initiation and the description of the degradation of material stiffness after the onset of necking was then realized using the Johnson–Cook failure model in combination with an appropriate damage evolution law.

#### 2.3.2. Numerical Damage Model

**Figure 2:**
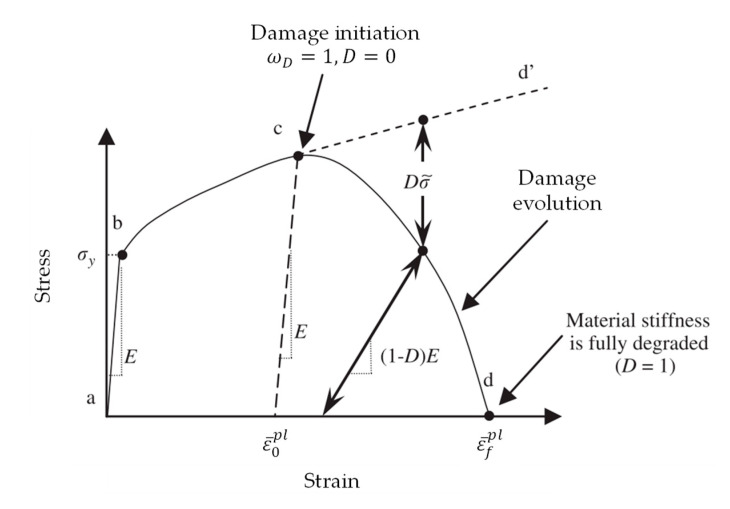
Typical uniaxial stress-strain response of a metal specimen in accordance to [35].

The numerical failure model was examined with nonlinear finite element simulations based on the conducted tensile tests using Abaqus CAE (6.14.5). Figure 2 shows a typical uniaxial stress-strain response of a ductile metal to illustrate the damage states. Initially, the ductile material response is linear elastic (a–b), characterized by the Young’s modulus, and followed by plastic yielding with strain hardening (b–c). Beyond the ultimate strength at point c there is a significant reduction in the load-bearing capacity (c–d) until fracture. Point c characterizes the material state where damage reaches a critical value and is coupled to the stress tensor, which is referred to as the damage initiation criterion. Beyond this point, the stress–strain response (c–d) is governed by the development of stiffness degradation, which is referred to as the damage evolution, up to the complete vanishing of the load-bearing capacity. In the context of damage mechanics, (c–d) can be viewed as the degraded response of the curve (c–d’) that the material would have followed in the absence of damage [34]. The effective material response (a-b-c-d’) is defined by the Young’s modulus, determined in tensile tests, and by the true stress-strain relation with Equations (1) and (2) up to the uniform elongation and beyond this point with Equation (3).

For damage initiation, the Johnson–Cook criterion implemented in Abaqus/Explicit was used [35]. It is a special case of ductile criterion, in which the equivalent plastic strain at the onset of damage, ${\bar{\varepsilon }}_{D}^{pl}$, is assumed to be of the form

(4) $${\bar{\varepsilon }}_{D}^{pl}=\left[d_{1}+d_{2}exp\left(-d_{3}\eta \right)\right]+\left[1+d_{4}ln\left(\frac{{\dot{\bar{\varepsilon }}}^{pl}}{{\dot{\varepsilon }}_{0}}\right)\right]\left[1+d_{5}\hat{\theta }\right]$$

where $d_{1}-d_{5}$ are failure parameters, ${\dot{\varepsilon }}_{0}$ is the reference strain rate, η= −p/q is the stress triaxiality, p is the hydrostatic pressure, q is the equivalent von Mises stress, ${\dot{\bar{\varepsilon }}}^{pl}$ is the equivalent plastic strain rate and $\hat{\theta }$ is the homologous temperature. Since the tensile tests were performed at room temperature and quasi-static load conditions, the constants $d_{4}$ und $d_{5}$ were neglected. The criterion for damage initiation is met when the following condition is satisfied:

(5) $$\omega_{D}=\int^{}\frac{d{\bar{\varepsilon }}^{pl}}{{\bar{\varepsilon }}_{D}^{pl}\left(\eta ,{\dot{\bar{\varepsilon }}}^{pl}\right)}=1$$

where $\omega_{D}$ is a state variable that increases monotonically with plastic deformations. The initial failure strain, ${\bar{\varepsilon }}_{0}^{pl}$, was determined in uniaxial tensile tests at η=1/3. Based on the literature [36,37], the failure parameters were determined in numerical simulations by varying $d_{1},\;d_{2}$ and $d_{3}$ until the equivalent plastic strain at the onset of damage matched the plastic strain of the experiments.

After damage initiation the material’s softening behavior can be observed, which is called damage evolution. Damage evolution was modelled by the overall damage variable *D*. In the case of D=0 the damage initiates to a finite element and final fracture occurs when *D* reaches 1. In this paper, the damage variable D was specified as a tabular function of the plastic displacement, ${\bar{u}}^{pl}$. The plastic displacement ${\bar{u}}^{pl}$ was defined using the evolution equation

(6) $${\dot{\bar{u}}}^{pl}=\{\begin{array}{c} 0\;\;\;\omega_{D}<1\;\\ L{\dot{\bar{\varepsilon }}}^{pl}\;\omega_{D}\geq 1\; \end{array}$$

where *L* is the characteristic length of the element. 

#### 2.3.3. Cyclic Behavior

The cyclic material curve was determined using multiple step tests (MST) and incremental step tests (IST). The reversal points of the stabilized stress-strain hysteresis were used to determine the cyclic stress-strain curve. For the analytical description of the stress-strain curve the approach of Ramberg and Osgood was used [38]. The cyclic stress-strain curve is written as follows:

(7) εa=εea+εpa=σaE+(σaK′)1n′

where *K*’ is the cyclic strength coefficient and *n*’ the cyclic strain-hardening exponent.

For the application of the strain-life approach the experimental strain-life data needs to be described by an analytical expression. According to Manson [39], Coffin [40] and Morrow [41], the relationship between strain amplitude and the number of load reversals can be expressed in the following form [42]

(8) $$\varepsilon_{a}=\varepsilon_{ea}+\varepsilon_{pa}=\frac{\sigma_{f}^{\prime }}{E}{\left(2N_{f}\right)}^{b}+\varepsilon_{f}^{\prime }{\left(2N_{f}\right)}^{c}$$

where, $\sigma_{f}^{\prime }$ and $\varepsilon_{f}^{\prime }$ are the fatigue strength and ductility coefficients, respectively. The exponents b and c are the fatigue strength and ductility exponents, respectively. 

## 3. Results and Discussion

### 3.1. Tensile Properties

#### 3.1.1. Stress-Strain Characteristics 

**Figure 3:**
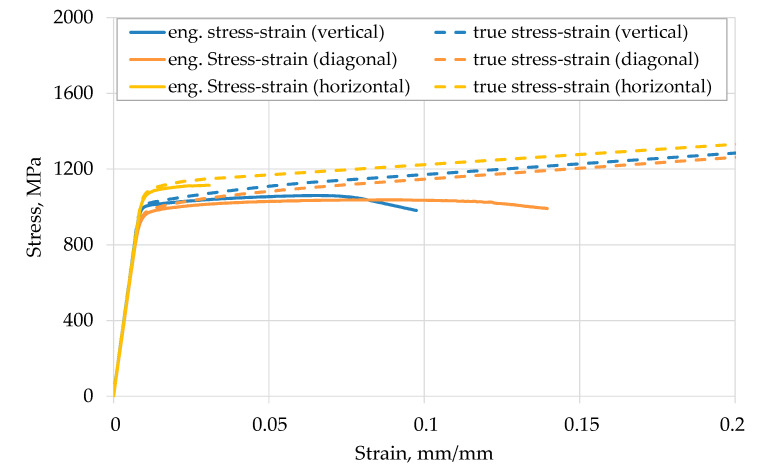
Engineering and true stress-strain curves from monotonic tensile test on EBM Ti-6Al-4V samples.

The results of the monotonic tensile tests of the EBM Ti-6Al-4V samples are presented in Figure 3 in terms of engineering stress-strain curves and true stress-strain curves. The shown engineering stress-strain curves are the average curves of the tested specimens. The true stress-strain curves were determined by Equations (1) and (2) up to the uniform elongation and beyond this point with Equation (3) using the mean engineering stress-strain curve. 

**Figure 4:**
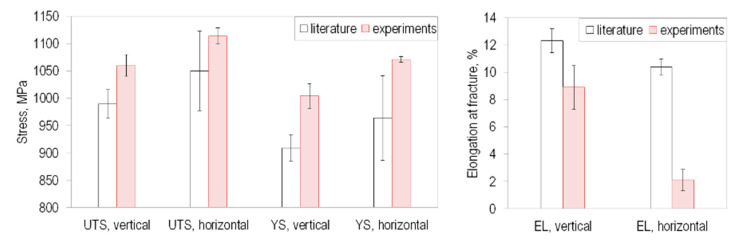
Experimentally determined tensile properties (ultimate tensile strength—UTS, yield strength—YS, elongation at fracture—EL) for vertically and horizontally built specimens compared to literature data of EBM manufactured Ti-6Al-4V machined specimens [8,25,26,27,28,44,45,46].

The evaluation of the mechanical tensile properties was carried out with an automated procedure in MATLAB (R20171b, MathWorks, Inc., Natick, MA, USA). The results are given as mean values ± standard deviation (SD) in Table 3. The Young’s moduli for the three investigated build directions differ only slightly from each other and are within the range of literature data [26,28]. The horizontally oriented specimens possess the highest strength, but the lowest elongation compared to vertically and diagonally built specimens. This trend is similar to the literature, as seen in Figure 4. However, the determined elongation at fracture of the horizontally built specimens is in the range of literature data with EBM tensile samples that were not post-machined [43]. However, a comparison of the experimental results with the literature shows that many influencing factors must be taken into account when evaluating the results. Even if the trend of the influence of the build direction is qualitatively verified, the quantitative values are influenced individually by each process. It is absolutely necessary to consider process conditions, e.g., process machine, process parameters and build direction. 

The strain distribution at the minimum cross-section becomes highly non-uniform when localized necking begins. Therefore, the local strain immediately before fracture is significantly larger than the measured elongation at fracture. One possibility for determining the fracture strain $\varepsilon_{f}$ is in an averaged sense to use the reduction of the cross-sectional area $R_{A}$ in the following analytical relation:

(9) $$R_{A}=1-e^{-\varepsilon_{f}}$$

The reduction in area was measured using the open source software ImageJ based on the microscopy images of the specimens’ cross sections at final fracture. The average reduction in area of the investigated specimens here was 31 ± 3% for the vertical build direction and 10 ± 3% for the horizontally built samples. Thus, the values for both directions are below the data published in the literature for EBM machined Ti-6Al-4V samples of 44.5% and 25% for vertically and horizontally built samples [25,27]. This again suggests that individual experiments are needed to characterize the EBM Ti-6Al-4V material. 

Another method, which was used to determine the fracture strain on the sample surface, was the DIC technique. For this, the major principle strain (e1) immediately prior to fracture was determined with DIC and listed with the analytically determined fracture strain with Equation (9) in Table 4. The difference is less than 10 percent for the two methods examined. However, numerical simulations are necessary to determine the true fracture strain. 

#### 3.1.2. Fracture Surfaces

**Figure 5:**
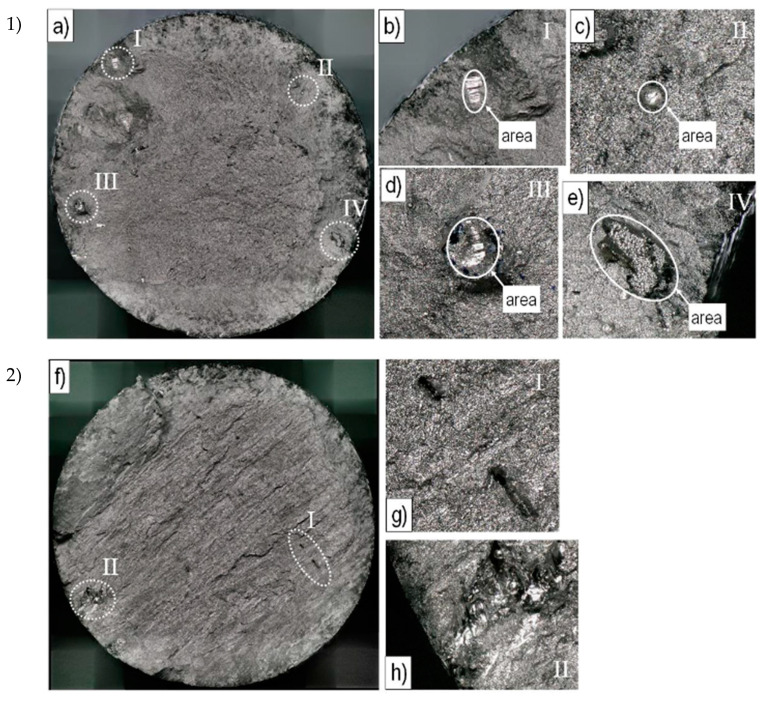
Different representative defect types in failed tensile samples for (**1**) the vertical and (**2**) diagonal build direction. Overview of the fracture surfaces in (**a**) and (**f**) with a magnification of defects in (**b**)–(**e**) and (**g**)–(**h**), respectively.

The fracture surfaces of the failed EBM Ti-6Al-4V tensile specimens show a cup-cone-shaped failure surface. Moreover, shear lips can be observed for all three build directions, which is representative of a ductile fracture mode. Two well-known EBM-related defect types, lack of fusions (LOF) and porosity, are also visible in the tensile samples examined. LOFs can be seen in Figure 5b,d,e,h, respectively. A porosity defect, caused by spherical gas pores, which can already be present in the initial powder, becomes obvious in Figure 5c. Both defects are present in samples of all build directions. However, the LOF defects occurred more frequently for specimens in the vertical build direction. Here, the defects are distributed over the entire fracture surface, whereas in the case of diagonally and horizontally manufactured specimens, the LOF defects occurred primarily at the surface or in contact to the surface.

**Figure 6:**
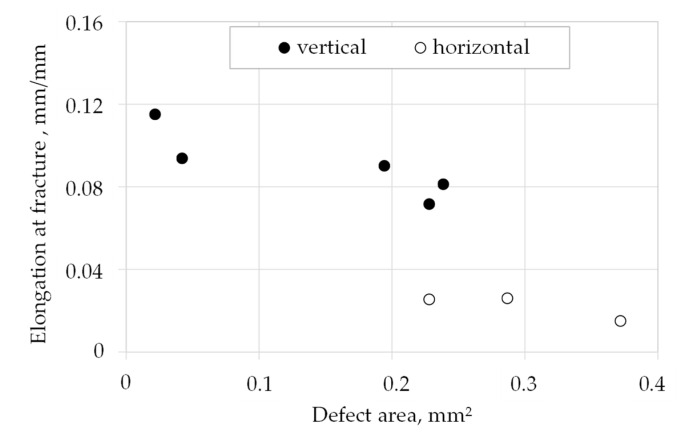
Correlation between defect area and elongation at fracture of vertically and horizontally built tensile specimens.

To determine the influence of the defect size on the elongation at fracture, the defect areas were measured. For this, the defect size was mostly approximated by a circle or ellipse around the defect area exemplarily shown in Figure 5b–e. If there were several defects on the surface, all defect areas were added together to form a total defect area. The results are shown in Figure 6 for vertically and horizontally built specimens. It can be seen that an increasing defect area results in decreased elongation at fracture. However, the defect area itself is not responsible for the elongation at fracture. This is because tensile samples built in vertical direction exhibit approximately 8% elongation with a defect area of approximately 0.2–0.25 mm^2^, which corresponds to 0.4%–0.5% of the fracture surface, whereas horizontally built samples exhibit only 3% elongation with the same defect area. Therefore, the size of the defect cannot be the only reason for reduced elongation. For example, the size, shape and location as well as the orientation of defects to the load direction should also be considered. 

#### 3.1.3. Damage and Failure Model

**Figure 7:**
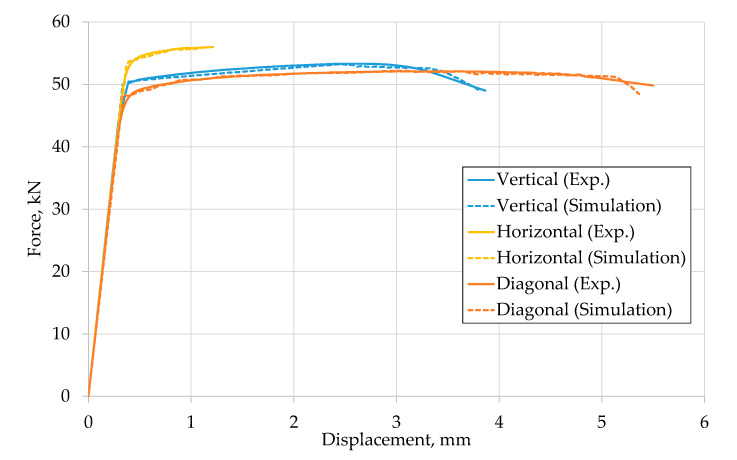
Comparison of force-displacement curves from experiments and numerical simulations.

Figure 7 shows the force-displacement curves obtained by monotonic tensile tests on EBM manufactured Ti-6Al-4V solid specimens with three investigated build directions in comparison to the appropriate numerical simulation results. The damage behavior was simulated with the damage initiation criterion by Johnson and Cook (Equation (4)). The determined J–C failure parameters are listed in Table 5.

The established tabular look-up of the state variable *D* and the plastic displacement ${\bar{u}}^{pl}$ for damage evolution are presented in Table 6. Therefore, the plastic displacements were determined from the simulations using the maximum true equivalent plastic strains in the interior of the fracture cross-section according to Equation (6). Due to the dependency of the plastic displacement on the characteristic element length, the used element length in the FEA must be considered. The ascertained damage evolutions were determined by an FE model with an element length of 0.3 mm. 

The elastic and plastic parts of the numerical curve are in good agreement with the experimental curve, as shown in Figure 7.

### 3.2. Fatigue Performance 

#### 3.2.1. Cyclic Stress-Strain Curves 

**Figure 8:**
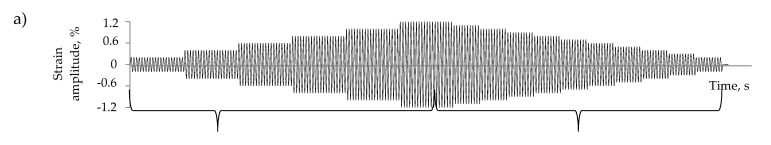

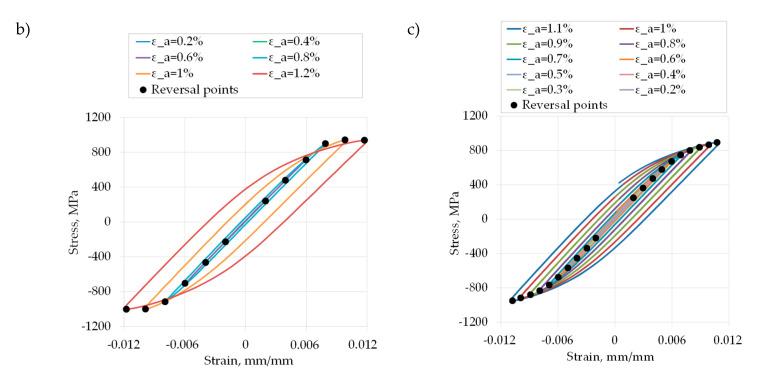
Determination of stress-strain plots from stabilized stress-strain hysteresis of (**a**) multiple step tests distinguished for (**b**) an ascending and (**c**) a descending part. The reversal points are used to determine the cyclic stress-strain curves.

The experimental program of the MST can be seen in Figure 8a. In these tests, an “ascending” part, which ranges from 0.2% to 1.2% strain amplitudes (Figure 8b) and a “descending” part, which ranges from 1.1% to 0.2% strain amplitude (Figure 8c) were considered separately. This was necessary to achieve an optimum fit between experimental and analytical data of the stress–strain data. Additionally, the compressive and tensile parts in the stress–strain plots were considered individually.

The reversal points of the stabilized stress-strain hystereses (black dots in stress-strain diagrams shown in Figure 8b,c) were used for the determination of the cyclic stress-strain curve according to Equation (7). The best fit of the analytically determined Ramberg–Osgood parameters is listed in Table 7. 

In general, the IST is a faster method compared to the MST for identifying cyclic stress–strain data. The results in Table 7 show, that the outcomes of the IST are comparable to the descending part of the MST. Moreover, in the ascending part of the MST the Ramberg–Osgood parameters are considerably lower than in the IST and the descending part of the MST. This is due to the fact that the material in the descending part of the MST and in the IST is plasticized, because the yield stresses were exceeded (Figure 8a). During the descending part of the MST, the material is pre-plasticized based on the preceding ascending part of the MST. Due to the diamond-shape during the IST the yield stress is recurrently exceeded. Against it, in the ascending part of the MST only at the end is the yield stress uniquely passed. However, the determined Young’s moduli are larger in the ascending part of the MST, because recognizable damage must have occurred in the descending part. In addition, no significant effect of the build direction on the determined Young’s moduli is observable.

**Figure 9:**
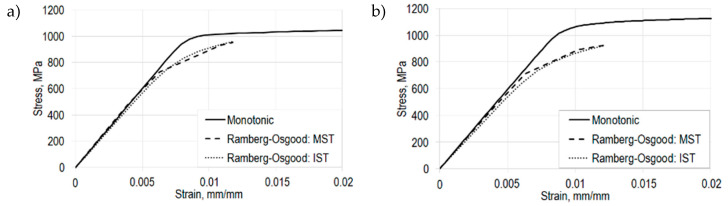
Softening behavior of Ti-6Al-4V for (**a**) vertically oriented specimens and (**b**) horizontally oriented specimens.

The comparison of the monotonic curves with the cyclic curves, determined from MST (descending, tensile) and IST (tensile part), indicates a softening behavior of EBM manufactured Ti-6Al-4V parts for the vertical and horizontal build directions (Figure 9a,b). This could already be seen in the ratio between the ultimate tensile strength and the yield strength for the vertically, diagonally and horizontally built specimens (Table 3), which are about 1.06, 1.08 and 1.04, respectively. All these ratios are less than 1.2, which is an indicator of the cyclic strain softening behavior of the investigated material [47].

#### 3.2.2. Strain-Life Curves 

**Figure 10:**
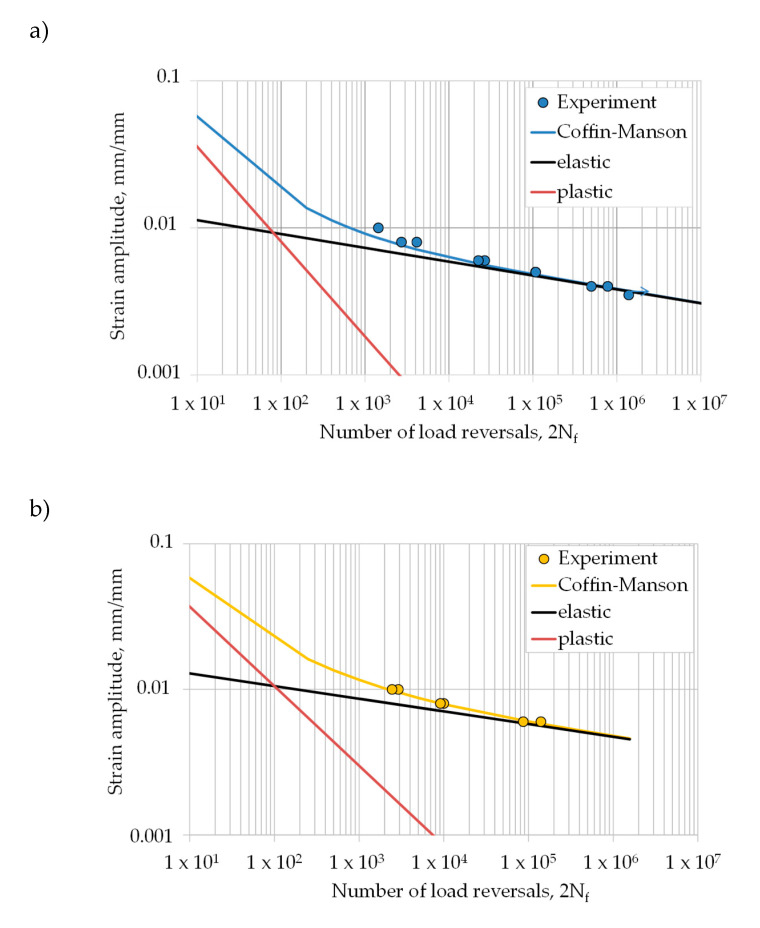
Experimental strain-life data fitted by Coffin-Manson for the investigated EBM Ti-6Al-4V specimens with (**a**) vertical and (**b**) horizontal build direction.

Figure 10 presents a plot of the strain amplitude, $\varepsilon_{a}$, versus reversals to failure, 2N_f_, for the fully-reversed strain-controlled fatigue tests generated in this study, as well as fitted curves determined with the Coffin-Manson Equation (8). The best fit of the Coffin–Manson parameters is listed in Table 8. It can be observed that the fatigue performance of horizontally built specimens is superior compared to the vertically built EBM Ti-6Al-4V parts.

#### 3.2.3. Fractography Analysis

**Figure 11:**
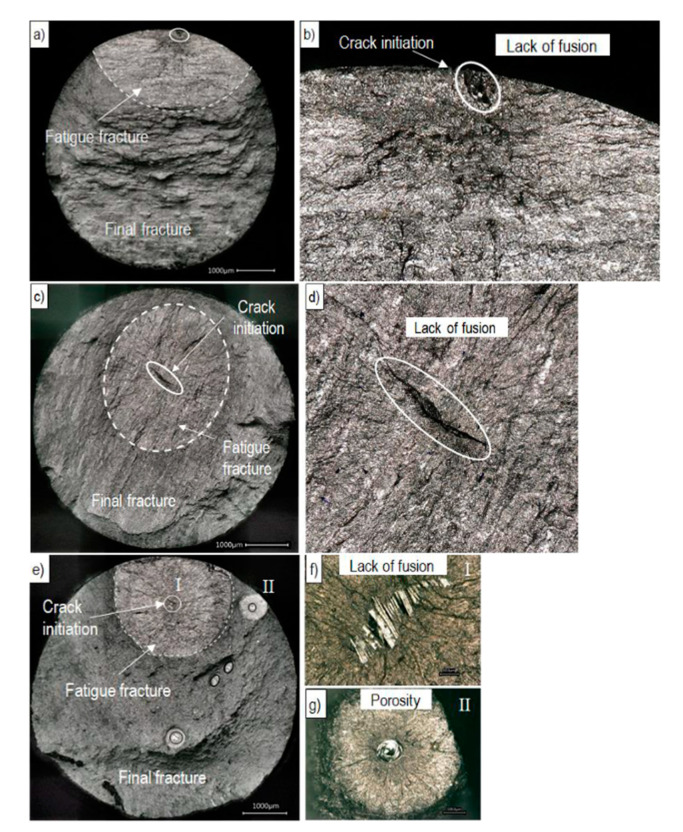
Comparison of fatigue fracture surfaces of EBM manufactured Ti-6Al-4V samples produced in (**a**)–(**d**) horizontally and (**e**)–(**g**) vertically build direction. The defects are shown in higher magnification on the right side.

Figure 11 shows exemplarily the fracture surfaces of investigated fatigue samples to reveal the reasons for crack initiation and crack propagation. Crack initiation sites, fatigue fracture as well as final fracture are highlighted. The failure was initiated either by lack of fusion defects or by porosity defects. Similar to the investigated tensile samples, the defect types occurred in both build directions. Further, the fractography analyses reveal that LOF defects are the primary crack initiation factor in all investigated EBM Ti-6Al-4V samples.

**Figure 12:**
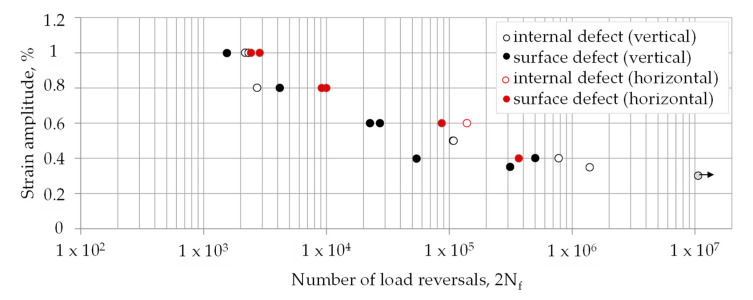
Strain-life data for horizontally and vertically built specimens.

Two different defect locations were distinguished at the fracture surfaces: (i) surface or in contact with the surface (sub-surface) (see Figure 11a) and (ii) internal defects (see Figure 11c,e). In Figure 12 the strain-life curves depending on the defect locations and the build direction are shown. For the horizontally built specimens only one internal defect initiation is visible, whereas in the vertically built specimens the initiation sites are almost equally distributed. Moreover, a difference between the data of vertically and horizontally built specimens is observable. 

The defect sizes were analyzed in terms of $\sqrt{area}$ (square root of the defect area projected onto the plane perpendicular to the maximum principal stress) in accordance with Murakami’s approach [16]. The defect sizes are in the range of approximately 120–300 µm for vertically and 75–200 µm for horizontally built specimens. In the following, Murakami’s approach was adapted to the strain-life approach. In the original form, Murakami’s approach allows to estimate a threshold stress for R = −1 based on the defect size in terms of $\sqrt{area}$ and the Vickers hardness. The influence of the defect position is considered since different relations for surface/subsurface and for internal defects are applied:

(10) $$\sigma_{W\sqrt{area}}=\frac{1,43\cdot \left(HV+120\right)}{{\left(\sqrt{area}\right)}^{\frac{1}{6}}}\;(\mathrm{for}\;\mathrm{surface}\;\mathrm{and}\;\mathrm{subsurface}\;\mathrm{defects})$$

(11) $$\sigma_{W\sqrt{area}}=\frac{1,56\cdot \left(HV+120\right)}{{\left(\sqrt{area}\right)}^{\frac{1}{6}}}\;(\mathrm{for}\;\mathrm{internal}\;\mathrm{defects})$$

For the vertically built specimens a value of HV_(vertical)_ = 348 and for the horizontally built specimens a value of HV_(horizontal)_ = 359 was determined. The first step in the adaption of Murakami’s approach was to convert the threshold stress $\sigma_{W\sqrt{area}}$ into a strain value. Since $\sigma_{W\sqrt{area}}$ is far below the yield stress Hooke’s law was used:

(12) $$\varepsilon_{W\sqrt{area}}=\frac{\sigma_{W\sqrt{area}}}{E}.$$

**Figure 13:**
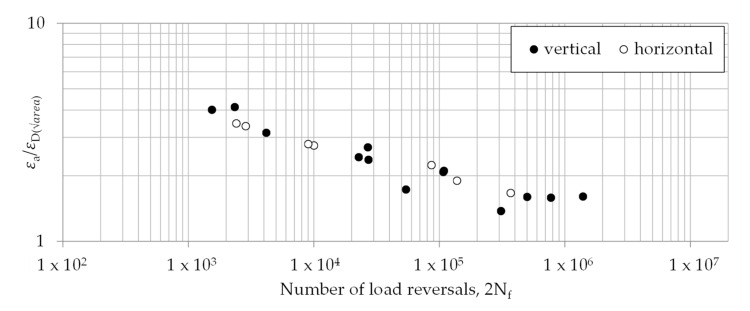
Modified strain-life data following an adapted Murakami’s approach.

In a second step, this threshold strain was used to calculate a modified strain-life curve using Murakami’s approach. Therefore, the relation between the applied strain and the threshold strain was determined and plotted versus the number of load reversals, as shown in Figure 13. It becomes obvious that the difference disappears between both build directions. These results suggest that the defects are the main influencing factor, rather than changes in the bulk material behavior. 

## 4. Summary and Conclusions

Additive manufacturing (AM) plays an important role in biomedical applications like load-bearing implants. These load-bearing implants are often quasi-static as well as cyclic loaded. For a safe design, a precise characterization of the cyclic and fatigue properties of the AM material is as important as its monotonic properties. In this context extensive experimental investigation of EBM manufactured Ti-6Al-4V samples, which is a preferred material for load-bearing implants, was carried out. The main conclusions are as follows:

1. The comparison of the experimental monotonic tensile test results with literature data shows that many influencing factors must be taken into account when evaluating the results. Even if the trend of the influence of the build direction is qualitatively verified, the quantitative values are influenced individually by each process. It is absolutely necessary to consider process conditions, e.g., process machine, process parameters and build direction.
2. For the description of the material behavior in the case of large plastic deformations, e.g., in damage models, the build direction must be taken into account, since it has a significant influence on the ductility.
3. The Johnson–Cook failure model, including the damage initiation and damage evolution law, is a suitable model to describe the EBM Ti-6Al-4V material behavior until failure.
4. While the defect size influences the elongation at fracture, it is not solely responsible for the elongation. For example, the build direction and the resulting size, shape and location of defects in relation to the load direction should also be considered.
5. To determine the cyclic stress–strain curve, multiple step tests and incremental step tests were performed. It could be observed that plasticization played an important role.
6. The strain-life curves of EBM Ti-6Al-4V can be described using the Coffin–Manson approach. The results show that a horizontal build direction leads to longer lifetimes compared to the vertical build direction.
7. In addition, Murakami´s approach was adapted to the strain-life approach, taking the defect size and position into account. These results suggest that the defects are the main influencing factor on the fatigue lifetime, rather than changes in the bulk material behavior.

## Figures and Tables

**Table 1:** Chemical compositions of Ti-6Al-4V ELI powders (wt%) [33].

(%)							
Ti	Al	V	C	Fe	O	N	H
Balance	6.0	4.0	0.03	0.1	0.1	0.01	<0.003

**Table 2:** Experimental program.

Build Direction	Tensile Test	Multiple Step Test		Incremental Step Test		Strain-Life Test	
	Number	Number	Strain Amplitudes	Number	Strain Amplitudes	Number	Strain Amplitudes
Vertical	8	4	0.2–1.2%	1	0.1–1.2%	14	0.3–1%
Diagonal	3	/	/	/	/	/	/
Horizontal	3	2	0.2–1.2%	1	0.1–1.2%	6	0.4–1%

**Table 3:** Monotonic tensile properties of EBM Ti-6Al-4V samples built in different orientations.

Build Direction	Young’s Modulus, GPa	Yield Strength, MPa	Ultimate Tensile Strength, MPa	Elongation at Fracture, %
Vertical	118 ± 1.3	1004 ± 22	1060 ± 20	8.9 ± 1.6
Diagonal	114 ± 0.4	957 ± 5	1037 ± 4	13.1 ± 1.3
Horizontal	115 ± 0.3	1071 ± 5	1114 ± 15	2.1 ± 0.8

**Table 4:** Fracture strain immediately prior to fracture determined with DIC (e(1)) and analytically determined with digital microscopy ($\varepsilon_{f}$).

Build Direction	e(1), %	ε_f_, %
Vertical	35.3 ± 5.5	37.7 ± 4.5
Diagonal	32.8 ± 3.1	29.9 ± 2.1
Horizontal	/	10.2 ± 3.6

**Table 5:** J–C failure parameters identified on tensile tests for different build directions.

	Vertical	Diagonal	Horizontal
$d_{1}$	0.04	0.08	0.02
$d_{2}$	0.025	0.04	0.01
$d_{3}$	−2.5	1.5	3.5

**Table 6:** Numerically determined damage evolution parameters for EBM Ti-6Al-4V specimens built in vertical, diagonal and horizontal direction.

Vertical		Diagonal		Horizontal	
*D*	${\bar{u}}^{pl}\;(\mathrm{mm})$	*D*	${\bar{u}}^{pl}\;(\mathrm{mm})$	*D*	${\bar{u}}^{pl}\;(\mathrm{mm})$
0	0	0	0	0	0
0.007	0.005	0.007	0.005	1	0.0006
0.011	0.02	0.011	0.04		
0.15	0.05	0.15	0.055		
1	0.06	1	0.06		

**Table 7:** Analytical description of the cyclic stress-strain curves determined from multiple step tests and incremental step tests for vertically and horizontally oriented specimens.

		MST—Ascending		MST—Descending		IST	
		Tensile	Compr.	Tensile	Compr.	Tensile	Compr.
K’, MPa	vertical	1023 ± 60	1090 ± 42	1648 ± 99	1578 ± 127	1700	1660
	horizontal	1020 ± 28	1010 ± 0	1400 ± 28	1750 ± 113	1710	1700
n’	vertical	0.012 ± 0.01	0.011 ± 0.003	0.1 ± 0.01	0.077 ± 0.01	0.098	0.081
	horizontal	0.017 ± 0.005	0.007 ± 0	0.075 ± 0.004	0.106 ± 0.01	0.11	0.095
E, GPa	vertical	122.6 ± 2.8	119.9 ± 3.3	114.2 ± 2.2	117.6 ± 4.4	114.6	116.9
	horizontal	119.9 ± 1.9	118.2 ± 2.3	110.6 ± 0.5	116.5 ± 0.8	108.8	109.4

**Table 8:** Analytical description of the strain-life curves for vertically and horizontally oriented specimens with parameters of the Coffin–Manson approach.

	$\sigma_{f}^{\prime },\;\mathrm{MPa}$	*b*	$\varepsilon_{f}^{\prime },\;\mathrm{mm}/\mathrm{mm}$	*c*
vertical	1722	−0.094	0.1575	−0.645
horizontal	1935	−0.087	0.13	−0.546
